# Multi-modality self-attention aware deep network for 3D biomedical segmentation

**DOI:** 10.1186/s12911-020-1109-0

**Published:** 2020-07-09

**Authors:** Xibin Jia, Yunfeng Liu, Zhenghan Yang, Dawei Yang

**Affiliations:** 1 Faculty of information technology, Beijing University of Technology, Beijing, China; 2 Department of Radiology, Beijing Friendship Hospital, Capital Medical University, Beijing, China

**Keywords:** Multi-modal fusion, Attention mechanism, 3D biomedical segmentation

## Abstract

**Background:**

Deep learning based on segmentation models have been gradually applied in biomedical images and achieved state-of-the-art performance for 3D biomedical segmentation. However, most of existing biomedical segmentation researches take account of the application cases with adapting a single type of medical images from the corresponding examining method. Considering of practical clinic application of the radiology examination for diseases, the multiple image examination methods are normally required for final diagnosis especially in some severe diseases like cancers. Therefore, by considering the cases of employing multi-modal images and exploring the effective multi-modality fusion based on deep networks, we do the research to make full use of complementary information of multi-modal images referring to the clinic experiences of radiologists in image analysis.

**Methods:**

Referring to the human radiologist diagnosis experience, we discuss and propose a new self-attention aware mechanism to improve the segmentation performance by paying the different attention on different modal images and different symptoms. Firstly, we propose a multi-path encoder and decoder deep network for 3D biomedical segmentation. Secondly, to leverage the complementary information among different modalities, we introduce a structure of attention mechanism called the Multi-Modality Self-Attention Aware (MMSA) convolution. Multi-modal images we used in the paper are different modalities of MR scanning images, which are input into the network separately. Then self-attention weight fusion of multi-modal features is performed with our proposed MMSA, which can adaptively adjust the fusion weights according to the learned contribution degree of different modalities and different features revealing the different symptoms from the labeled data.

**Results:**

Experiments have been done on the public competition dataset BRATS-2015. The results show that our proposed method achieves dice scores of 0.8726, 0.6563, 0.8313 for the whole tumor, the tumor core and the enhancing tumor core, respectively. Comparing with the U-Net with SE block, the scores are increased by 0.0212,0.031,0.0304.

**Conclusions:**

We present a multi-modality self-attention aware convolution, which have better segmentation results based on the adaptive weighting fusion mechanism with exploiting the multiple medical image modalities. Experimental results demonstrate the effectiveness of our method and prominent application in the multi-modality fusion based medical image analysis.

## Background

Medical image segmentation is one of the most common areas of applying deep learning into the medical image analysis. Meanwhile, semantic segmentation is usually under the request to do the automatic partition of the interesting areas such as organs and lesions, which will be applied in the assistant diagnosis [1], the tissue-specific measurement [2], the three-dimensional reconstruction [3], and the visual enhancement [4].

Traditional image segmentation methods include threshold-based [5], deformable surface modal based [6], active surface modal based [7], etc. The performance of these methods is limited, for the reason of similarity between interested areas and surroundings. Moreover, determining interested areas usually strongly depends on handcrafted features that suffer from the limited feature representation ability [8]. Deep learning is constantly creating new achievements in computer vision and pattern recognition. In some tasks of natural image classification, the performance of deep learning based on approaches even surpasses that of the human judgment [9]. The achieved good performances of state-of-the-art deep learning techniques are mainly attributed to the ability of the convolutional neural network (CNN) to learn the hierarchical representation of images, so that it does not depend on the handcrafted features and overcomes the limitation of handcrafted features in revealing the characteristics of complex objects [10]. The strong feature learning ability of CNN opens up a new direction for medical image segmentation. CNN is typically used for classification, and the output of images is mostly only the category labels. In the task of medical image segmentation, the desired output should include location, that is, the classification of each pixel is necessary. Patch-based method [11–13] determines the class of each pixel by predicting the label of the local area around each pixel (by using sliding window method), However, the training of this method is very slow and it is difficult to determine the most appropriate size of the local area. The larger area will affect the accuracy, while the smaller area is difficult to consider the context information. Fully convolution networks (FCNs) [14] solves these two problems efficiently and elegantly. Unlike classical CNN, which uses fully connection layer to get fixed-length vectors after the convolution layer for classification, FCN uses deconvolution to up-sample the feature map and restore it to the same size as the input image, thus each pixel can be predicted. On this basis, U-Net [15] designs the network structure consisting of an encoder path that contains multiple convolutions for down sampling, and a decoder path that has several deconvolution layers to up-sample the feature. Furthermore, it combines high-resolution features with up-sampled features by using skip connection to improve positioning accuracy. This encoder-decoder structure has also become the basic structure of many segmentation method,including segmentation of 3D medical images that can make better use of depth information [16–19]. However, due to the indistinguishability of interested areas in tissues, for example, the tumors with the surrounding normal tissues, it is still a big challenge to establish effective methods for medical image semantic segmentation.

Referring to the clinic diagnosis experience of radiologists, the diagnosis report is made based on synthesizing multiple-perspective clues from the multiple medical imaging methods. For example, four different modes of MRI (Magnetic Resonance Imaging) images are used in brain tumor surgery: T1 (spin-lattice relaxation), T1c (T1-contrasted), T2 (spin-spin relaxation), and Flair (fluid attenuation inversion recovery). Enhancing and non-enhancing structures are segmented by evaluating the hyper-intensities in T1C. T2 highlights the edema and Flair is used to cross-check the extension of the edema. Each modality has distinct responses for different sub regions of gliomas. The final diagnosis is usually determined by multiple modalities. Because the information provided by single modal images is very limited, it is difficult to meet the high-precision clinical needs. Multi-modal images provide more information about the patient’s lesion and its surrounding areas, and the information of different modalities is complementary each other in revealing the lesion characteristics from different perspectives. How to make good use of these complementary information has become a direction to improve the accuracy of segmentation. Existing methods often treat modalities as different channels in the input data [20, 21]. However, the correlations between them are not well utilized. To draw inspiration from the recent success of SKNet [22] and understanding of clinic diagnosis experience, we propose a multi-modality self-attention aware deep network for 3D biomedical segmentation. By using Multi-Modality Self-Attention Aware convolution to realize the self-weighted fusion of multi-modal data, it achieves state-of-the-art performance for multi-modal brain tumor segmentation.

## Methods

### Multi-path encoder and decoder

**Fig. 1:**
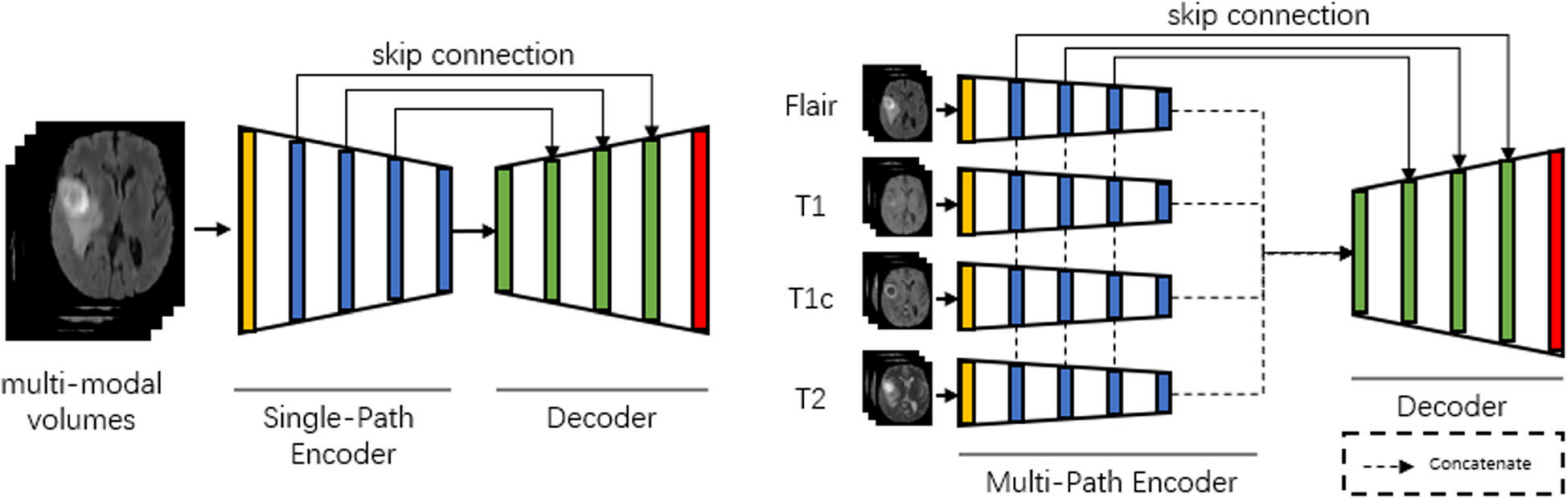
Comparisons of encoder and decoder structures

To realize processing of multi-modal 3D medical images, we explore to construct a multi-path input 3D segmentation network. The network adopted in the paper is the encoder and decoder structure similar to U-Net as shown in Fig. 1. Here, the encoder is used to extract the deep representation of each modality of medical image, while the decoder is used to up-sample the learned feature map at each level and restore feature at the last level to the original resolution for the pixel-wise region and semantic label prediction.

To deal with the multi-modal data at the encoder end, there are usually two solution: single-path with concatenating the multi¬-model image at the data-level and multi-path with concatenating the multi-modal image at the feature level. The structure of two fusion methods as illustrated in Fig. 1. Because the multi-path structure facilitates the processing of information from each modal separately, we take the multi-path as the base structure of the encoder.

More specifically, at the encoder end, we adopt a ResNet [23] as backbone network which consists of one input layer and four down-sampling layers. 3D convolution of the kernel size 3 × 3 and 7 × 7 is used for input and down-sampling layers respectively.

The structure of the decoder corresponds to the encoder, which includes four up-sampling layers and one output layer. For up-sampling layers, each 3D Transpose convolution with kernel size 3 × 3 is used to up-sampling feature map, and combines with the corresponding high-resolution features. All convolutions above are further applied by an element-wise rectified-linear non-linearity (ReLU). After up-sampling the feature maps to the original resolution, 1 × 1 convolution is used to produce the class probabilities of each pixel.

Referring to the experience of radiologists in clinical diagnosis based on overall consideration of significant symptoms reflecting on certain multiple modal images, we discuss an attention mechanism to improve the segmentation performance by paying the different attention on different features and different modal images. A new self-attention aware mechanism is proposed and illustrated in the following section.

### Multi-modality self-attention aware convolution

**Fig. 2:**
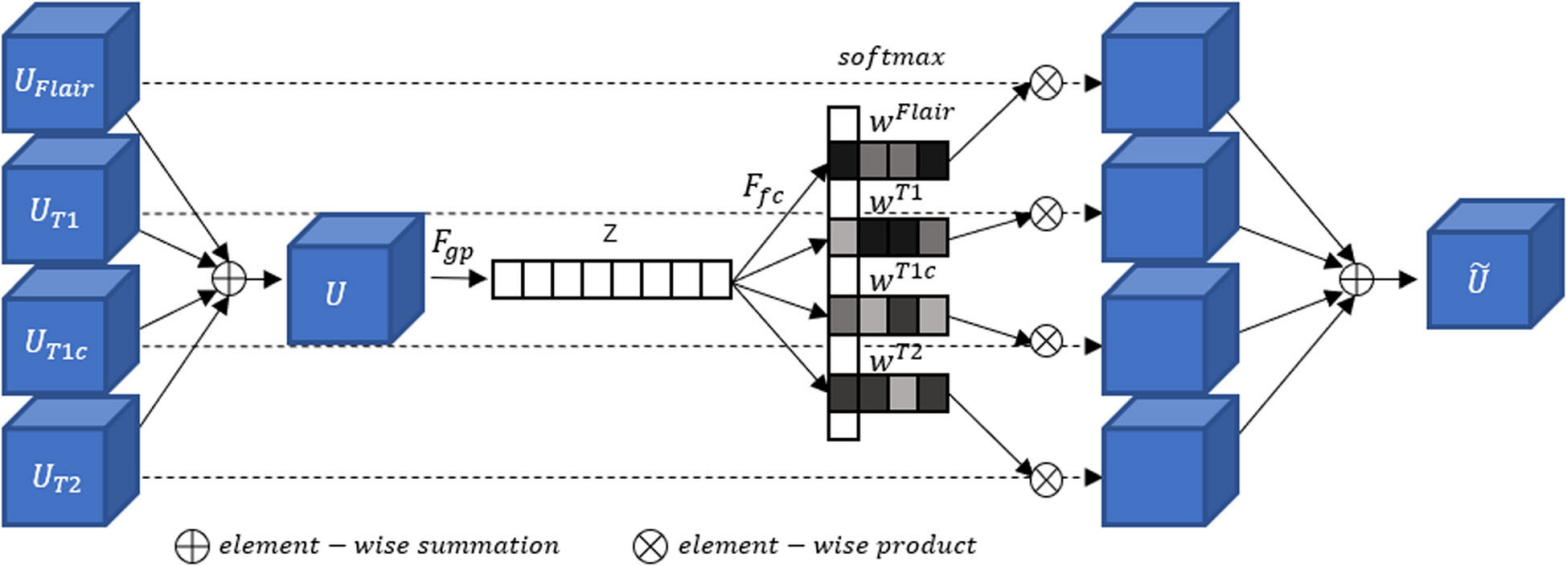
Multi-Modality Self-Attention Aware Convolution

Recently, attention mechanism is used for a series of tasks [24, 25], it biases the allocation of the most informative feature expressions and simultaneously suppresses the less useful ones. Furthermore, SENet [26] brings a gating mechanism to self-recalibrate the feature map via channel-wise importance. Then on the base of these, SKNet was proposed to focus on the adaptive local receptive fields size of neurons sizes. Similarly, we propose the Multi-Modality Self-Attention Aware Convolution to fuse multi-modal features, which can adaptively adjust the fusion weights according to the contribution degree of different modalities (see Fig. 2).

For the obtained multi-modal features *U*_*m*_ ∈ *R*^*W* × *H* × *D* × *C*^, we first fuse them via an element-wise summation to integrate information:

1 $$ \mathrm{U}={\sum}_m^M{U}_m $$

where W, H, D are feature dimensions, C is the number of channels and m is modality in M (all modalities).

Then we shrink each feature map on the channel by 3D global average pooling to generate channel-wise statistics as z ∈ *R*^*C*^. Specifically, the c-th element of z is calculated as:

2 $$ {z}_c={F}_{gp}\left({U}_c\right)=\frac{1}{\mathrm{W}\times \mathrm{H}\times \mathrm{D}}{\sum}_{i=1}^W{\sum}_{j=1}^H{\sum}_{k=1}^D{U}_c\left(i,j,k\right) $$

To realize the adaptive weighting of the multi-modal feature map, M full-connection layers are used to generate M weighting parameters w ∈ *R*^*C*^ under the guidance of feature descriptor z. Specifically, a SoftMax operator is applied on the channel-wise digits to adaptively select different modality of information:

3 $$ {\mathrm{w}}_c^m=\frac{e^{z_c^m}}{\sum_m^M{e}^{z_c^m}},{\sum}_m^M{\mathrm{w}}_c^m=1 $$

The final feature map $\overset{\sim }{U}\in {R}^{W\times H\times D\times C}$ is obtained through the attention weights between multi-modal:

4 $$ \overset{\sim }{U}={\sum}_m^M{w}_m\bullet {U}_m $$

**Fig. 3:**
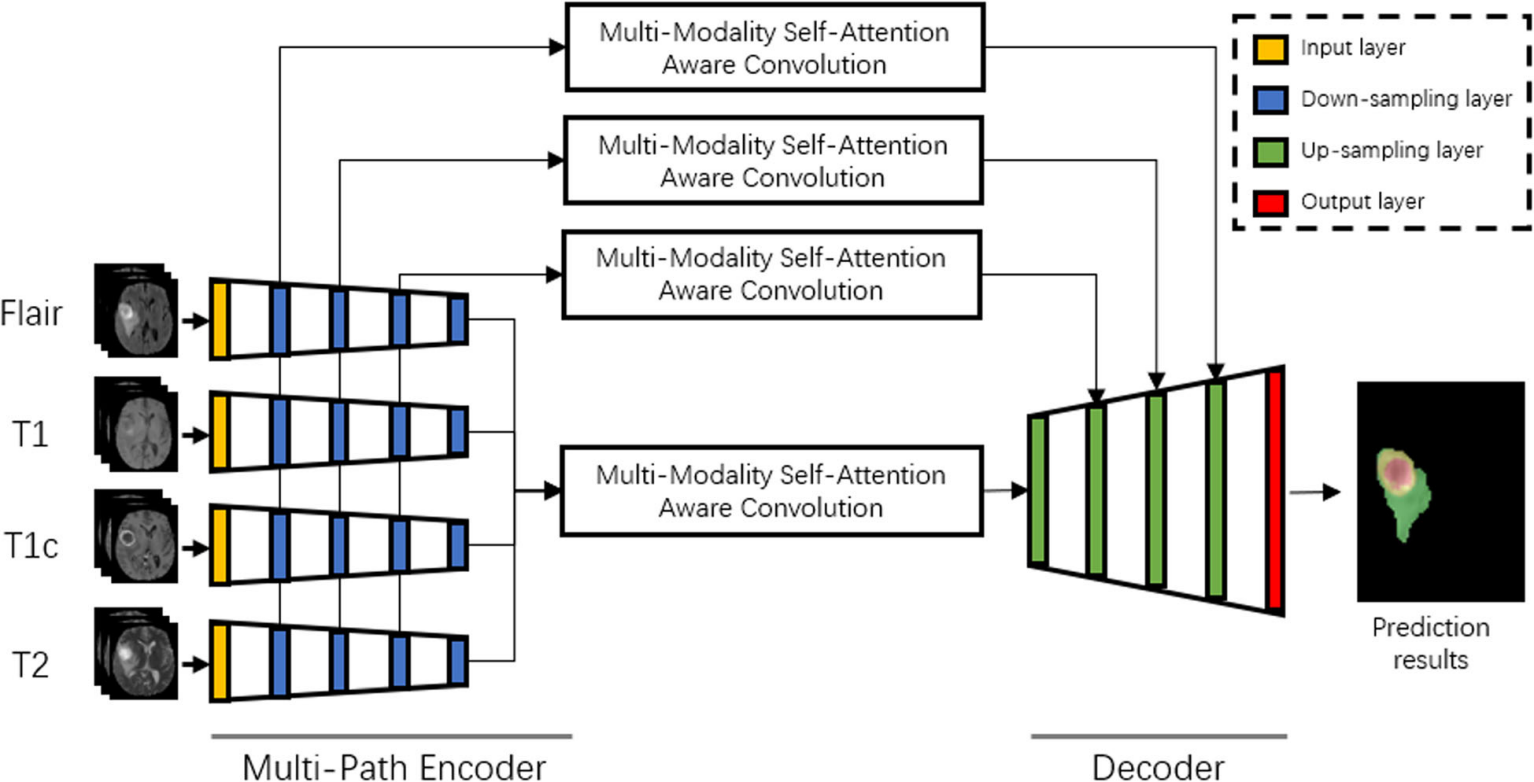
Multi-Modality Self-Attention Aware Deep Network

The system overview of our method shows in Fig. 3.

## Results

### Dataset and data preprocessing

The Dataset for this study comes from BRATS-2015 [27]. The training set consists of 220 patients with high grade gliomas and 54 subjects with low grade gliomas. The testing set contains images of 110 patients. Each patient was scanned with four sequences: T1, T1c, T2, and FLAIR. The size of each MRI image is 155 × 240 × 240. All of the images were skull-striped and re-sampled to an isotropic 1 mm3 resolution, and four sequences of the same patient had been co-registered. All ground truth annotations were carefully prepared under the supervision of expert radiologists. The ground truth contains five labels: non-tumor, necrosis, edema, non-enhancing tumor and enhancing tumor. Because the original testing set is without ground truth, we split the training data into two parts: 195 high grade gliomas and 49 low grade gliomas for training, and the rest 30 subjects for testing. For data preprocessing, we first extract the region of interest area from the original image to prevent the model from focusing on zero regions and getting trapped into a local minimum. Then we resize a volume to 144 × 144 for each axial plane and normalize the intensity of a volume based on the mean and standard error (std).

The evaluation was done for three different tumor sub-compartments:

- Enhancing Tumor (ET): it only takes the active tumor region (label 4 for high-grade only)
- Whole Tumor (WT): it considers all tumor areas (labels 1, 2, 3, 4)
- Tumor Core (TC): it considers tumor core region without necrosis (labels 1, 3, 4)

### Training set

The training patch size was 144 × 144 × 16 which means that we put 16 slices of volume into the network at a time. Our networks were implemented in Pytorch. We use stochastic gradient descent (SGD) optimizer for training, with the initial learning rate is 10e-3, momentum 0.9, weight decay 5 × 10^−4^, batch size 1 and maximal iteration 400. Network parameters are initialized by kaiming initialization. The Cross-Entropy loss plus Dice loss is used for training.

### Evaluation criteria

There are three kinds of Metrics in biomedical segmentation: Dice, Sensitivity, and Positive Predicted Value.

5 $$ Dice=\frac{2\ast TP}{\left(2\ast TP+ FP+ FN\right)} $$

6 $$ Sensitivity=\frac{TP}{\mathrm{TP}+\mathrm{FN}} $$

7 $$ Positive\ Predicted\ Value=\frac{TP}{\mathrm{TP}+\mathrm{FP}} $$

where TP, TN, FP, FN are meant as true positives, true negatives, false positives, and false negatives. Dice (Dice Similarity Index) is a measure of how similar both prediction and ground truth are. A high Sensitivity implies the most lesions were segmented successfully. Positive Predicted Value indicates the capability of a test to detect the presence of disease.

### Experimental results

In Table 1, we compare the performance of single-path with multi-path encoder by using a simple structure (shown in Fig. 1) on the testing set. The results show that the single-path encoder can make better use of multi-modal information, because the combination of input data in the channel dimension can make the convolution kernel of the encoder layers learn multi-modal information simultaneously and integrate it. Although the simple multi-path input cannot learn the complementary information of the multi-modal data, sharing parameters can solve this problem to a certain extent.

**Table 1:** Comparison of segmentation results between single and multi-path encoder

	Dice			Sensitivity			Positive Predicted Value		
	WT	TC	ET	WT	TC	ET	WT	TC	ET
Single	0.8135	0.5900	0.7687	0.8451	0.6732	0.8003	0.8651	0.6751	0.8164
Multi (unshared)	0.7912	0.5338	0.6833	0.7999	0.6557	0.7591	0.8635	0.5723	0.7395
Multi (shared)	0.8063	0.5777	0.7503	0.8414	0.6192	0.7951	0.8557	0.6007	0.7820

In Table 2, we compare the performance of two attention mechanisms. On the basis of the previous experiment, we added SE block [26] to each convolution layer to weight the multi-modal information on the channel dimension for the U-Net [15] structure. Then, we add our MMSA structure to the multi-path structure to realize the self-weighted fusion of multi-modal information. Experimental results show that both attention mechanisms can improve the performance of the original network, furthermore, our method achieves the optimal results.

**Table 2:** Segmentation result of two attention mechanisms

	Dice			Sensitivity			Positive Predicted Value		
	WT	TC	ET	WT	TC	ET	WT	TC	ET
U-Net + SE	0.8514	0.6253	0.8009	0.8666	0.7163	0.8599	0.8811	0.6624	0.8373
Multi (shared) + MMSA	0.8726	0.6563	0.8313	0.8695	0.7207	0.8613	0.8994	0.6738	0.8421

**Fig. 4:**
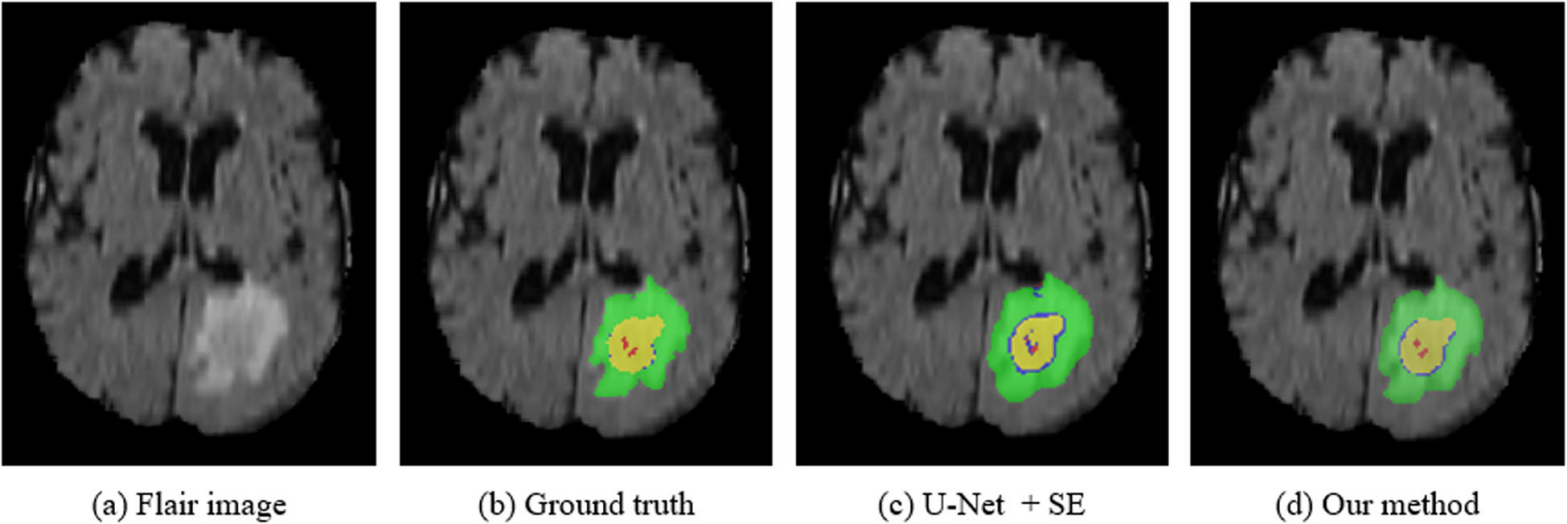
Segmentation result of the brain tumor from a training image

Figure 4 shows examples of segmentation results. For simplicity of visualization, only Flair image is shown. Among them, different colors represent different categories, green for edema, red for necrosis, yellow for enhancing tumor core, blue for non-enhancing tumor core. As shown in Fig. 4, our method is more accurate for the segmentation of lesions, and the area of misclassification is less comparing with the approach of single path with the SE block. The segmentation results are more approximate with that of the Ground truth.

## Discussion

In the independent testing set, the model obtains similar results. It shows that the model has a certain generalization ability in the task of glioma segmentation. In order to verify the effectiveness of self-attention aware convolution, the comparative experiment is carried out under the same training parameters. The starting point of this paper is to study how to make better use of multi-modal data. The task of brain glioma segmentation here is just to verify the performance of the model, and the method can be used for other multi-modal image segmentation tasks.

In order to cooperate with the multi-modal data fusion scheme proposed, we adopt the design of multi-path input. Therefore, missing modality and the change of input order will seriously affect the test results, which makes the model not flexible enough in use.

## Conclusions

In this paper, we introduce an attention mechanism architecture for 3D multi-modal image biomedical segmentation. With the proposed multi-modality self-attention aware convolution, the segmentation result is improved by counting the different impact of different features from different modalities. The self-attention aware deep network provides an effective solution for the multi-modal problem with adaptive weighting and fusion mechanism based on data learning. Experimental results on BRATS-2015 dataset demonstrate that our method is effective and achieves better segmentation results comparing the single path with simple concatenative and without taking account of the variety of each modality. In the future, more research with our proposed MMSA network will be done on the application of medical segmentation based on multi-parameter MRI in some complex application situation such as the liver diagnosis, where there exists close appearance among lesions and surroundings, at the meantime, large diversity exists among same types of lesions.

## Data Availability

The datasets analyzed during the current study are available in the SICAS Medical Image Repository, https://www.smir.ch/BRATS/Start2015

## Abbreviations

MMSA: Multi-Modality Self-Attention Aware
MR: Magnetic Resonance
CNN: Convolutional neural network
FCN: Fully convolution network
T1: Spin-lattice relaxation
T1c: T1-contrasted
T2: Spin-spin relaxation
Flair: Fluid attenuation inversion recovery
SKNet: Selective Kernel Network
ResNet: Residual Network
ReLU: Rectified-linear non-linearity
SENet: Squeeze-and-Excitation Network
SGD: Stochastic gradient descent

## References

[1] Porter CR, Crawford ED (2003). Combining artificial neural networks and transrectal ultrasound in the diagnosis of prostate cancer. ONCOLOGY-WILLISTON PARK THEN HUNTINGTON THE MELVILLE NEW YORK.

[2] Etoz S, Brace C L: Tissue Permittivity Measurement with Concurrent CT Imaging: Analysis of Heterogeneity Effects. IEEE 13th European Conference on Antennas and Propagation (EuCAP) 2019, 1–5.

[3] Chen Y, Sun P. The research and practice of medical image enhancement and 3D reconstruction system. IEEE International Conference on Robots & Intelligent System (ICRIS). 2017:350–3.

[4] Moradi M, Abolmaesumi P, Siemens DR (2008). Augmenting detection of prostate cancer in transrectal ultrasound images using SVM and RF time series. IEEE Trans Biomed Eng.

[5] Simonyan K, Zisserman A: Very deep convolutional networks for large-scale image recognition. arXiv preprint arXiv:1409.1556, 2014.

[6] Dosovitskiy A, Springenberg JT, Riedmiller M, et al. Discriminative unsupervised feature learning with convolutional neural networks. Adv Neural Inf Proces Syst. 2014:766–74. 10.1109/TPAMI.2015.2496141 26540673

[7] Otsu N (1979). A threshold selection method from gray-level histograms. IEEE Transactions Systems Man Cybernetics.

[8] Aboutanos GB, Nikanne J, Watkins N (1999). Model creation and deformation for the automatic segmentation of the brain in MR images. IEEE Trans Biomed Eng.

[9] Kobashi S, Fujimoto Y, Ogawa M, et al: Fuzzy-ASM based automated skull stripping method from infantile brain MR images. IEEE International Conference on Granular Computing (GRC 2007) 2007, 632–632.

[10] Chen Y, Juttukonda M, Lee Y Z, et al: MRI based attenuation correction for PET/MRI via MRF segmentation and sparse regression estimated CT. IEEE 11th International Symposium on Biomedical Imaging (ISBI) 2014, 1364–1367.

[11] Ciresan D, Giusti A, Gambardella LM, et al. Deep neural networks segment neuronal membranes in electron microscopy images. Adv Neural Inf Proces Syst. 2012:2843–51.

[12] Havaei M, Davy A, Warde-Farley D (2017). Brain tumor segmentation with deep neural networks. Med Image Anal.

[13] Pereira S, Pinto A, Alves V (2016). Brain tumor segmentation using convolutional neural networks in MRI images. IEEE Trans Med Imaging.

[14] Long J, Shelhamer E, Darrell T. Fully convolutional networks for semantic segmentation. Proc IEEE Conf Comput Vis Pattern Recognit. 2015:3431–40. 10.1109/TPAMI.2016.2572683 27244717

[15] Ronneberger O, Fischer P, Brox T: U-net: convolutional networks for biomedical image segmentation. International Conference on Medical image computing and computer-assisted intervention. Springer, Cham 2015, 234–241.

[16] Milletari F, Navab N, Ahmadi S A: V-net: Fully convolutional neural networks for volumetric medical image segmentation. IEEE Fourth International Conference on 3D Vision (3DV) 2016, 565–571.

[17] Kamnitsas K, Ledig C, Newcombe VFJ (2017). Efficient multi-scale 3D CNN with fully connected CRF for accurate brain lesion segmentation. Med Image Anal.

[18] Li W, Wang G, Fidon L, et al: On the compactness, efficiency, and representation of 3D convolutional networks: brain parcellation as a pretext task. International Conference on Information Processing in Medical Imaging. Springer, Cham 2017, 348–360.

[19] Stollenga MF, Byeon W, Liwicki M, et al. Parallel multi-dimensional LSTM, with application to fast biomedical volumetric image segmentation. Adv Neural Inf Proces Syst. 2015:2998–3006.

[20] Dolz J, Gopinath K, Yuan J (2018). HyperDense-net: a hyper-densely connected CNN for multi-modal image segmentation. IEEE Trans Med Imaging.

[21] Wang G, Li W, Ourselin S, et al: Automatic brain tumor segmentation using cascaded anisotropic convolutional neural networks. International MICCAI Brain lesion Workshop. Springer, Cham 2017, 178–190.

[22] Li X, Wang W, Hu X, et al. Selective kernel networks. Proc IEEE Conf Comput Vis Pattern Recognit. 2019:510–9.

[23] He K, Zhang X, Ren S, et al: Identity mappings in deep residual networks. European conference on computer vision. Springer, Cham 2016, 630–645.

[24] Chen D, Zhang S, Ouyang W, et al. Person search via a mask-guided two-stream cnn model. Proceedings European Conference Computer Vision (ECCV). 2018:734–50.

[25] Tian W, Wang Z, Shen H, et al: Learning better features for face detection with feature fusion and segmentation supervision. arXiv preprint arXiv:1811.08557, 2018.

[26] Hu J, Shen L, Sun G. Squeeze-and-excitation networks. Proc IEEE Conf Comput Vis Pattern Recognit. 2018:7132–41.

[27] Menze BH, Jakab A, Bauer S (2014). The multimodal brain tumor image segmentation benchmark (BRATS). IEEE Trans Med Imaging.

